# Chromatic Induction in Migraine

**DOI:** 10.3390/vision5030037

**Published:** 2021-08-06

**Authors:** Xim Cerda-Company, Olivier Penacchio, Xavier Otazu

**Affiliations:** 1 Computer Vision Center, Computer Science Department, Universitat Autònoma de Barcelona, Bellaterra, 08193 Barcelona, Spain; xcerda@cvc.uab.cat; 2 School of Psychology and Neuroscience, University of St Andrews, St Andrews KY16 9JP, UK; op5@st-andrews.ac.uk

**Keywords:** migraine, vision, colour, colour perception, chromatic induction, psychophysics

## Abstract

The human visual system is not a colorimeter. The perceived colour of a region does not only depend on its colour spectrum, but also on the colour spectra and geometric arrangement of neighbouring regions, a phenomenon called chromatic induction. Chromatic induction is thought to be driven by lateral interactions: the activity of a central neuron is modified by stimuli outside its classical receptive field through excitatory–inhibitory mechanisms. As there is growing evidence of an excitation/inhibition imbalance in migraine, we compared chromatic induction in migraine and control groups. As hypothesised, we found a difference in the strength of induction between the two groups, with stronger induction effects in migraine. On the other hand, given the increased prevalence of visual phenomena in migraine with aura, we also hypothesised that the difference between migraine and control would be more important in migraine with aura than in migraine without aura. Our experiments did not support this hypothesis. Taken together, our results suggest a link between excitation/inhibition imbalance and increased induction effects.

## 1. Introduction

**Figure 1:**
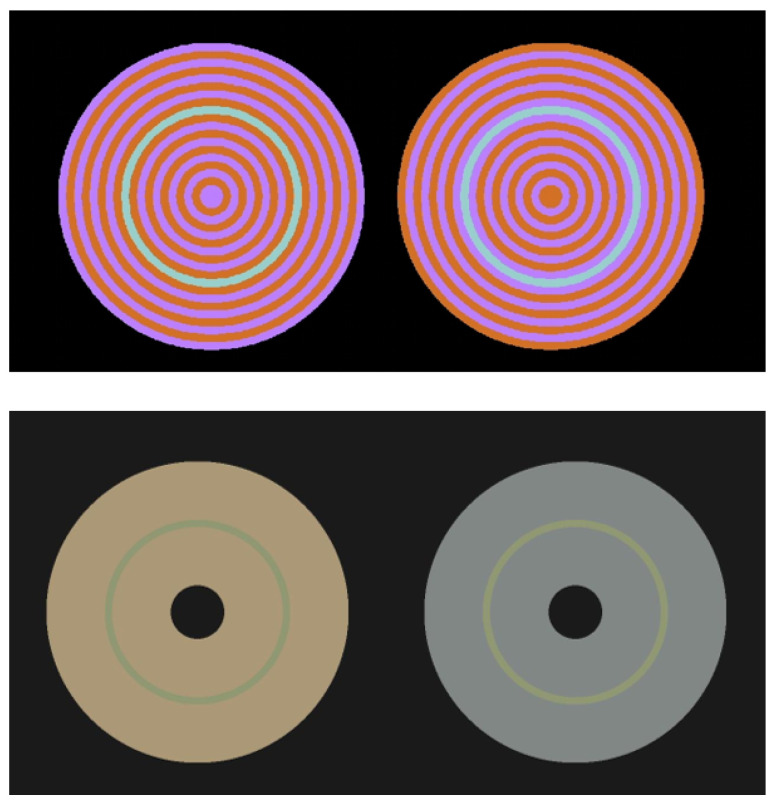
Examples of colour assimilation (**top** panels) and colour contrast (**bottom** panels). **Top** panel: On the left side a bluish-green ring is surrounded by alternate magenta (first inducer, in contact with the bluish-green ring) and purple (second inducer) rings. On the right side, the same bluish-green ring is surrounded by the same inducers in reverse order. In spite of having the same physical colour (i.e., reflectance), these two rings are, respectively, perceived as green and blue, an effect referred to as colour assimilation because the colour of the ring shifts towards that of the first inducer (the bluish-green ring becomes greener when in contact with the magenta ring and more bluish when in contact with the purple ring). **Bottom** panel: example of colour contrast, where the colour of the green-lime ring shifts away from the colour of a unique inducer (left, a brown disk; right, a grey disk). On the left, the colour of the green-lime ring shifts away from the brown of the disk and is perceived as greenish. On the right, it shifts away from the grey ring and is perceived as closer to lime.

Chromatic induction refers to the change in the perceived colour of a region caused by the colour(s) and spatial configuration of the surrounding regions, called inducers. There are two types of chromatic induction: assimilation and contrast. Assimilation occurs when the colour of a central region shifts towards the colour of an inducer, as illustrated in the top panels of Figure 1. Contrast occurs when the colour of a central region shifts away from the colour of an inducer [1], as illustrated in the bottom panels of Figure 1. Surrounding regions with a uniform colour usually cause chromatic contrast while surrounding regions made of stripes with different colours usually cause assimilation [2,3,4].

The mechanisms underlying chromatic induction are still poorly understood [4,5,6]. Nevertheless, experimental evidence suggests that centre-surround mechanisms and lateral connections play a central role in induction effects. Lateral connections consist of an interplay between excitatory and inhibitory connections that allow the activity of a neuron to be modified by the activity of other neurons beyond its classical receptive field [7,8,9,10]. The activity of a neuron responding to colour in a central region can therefore be modified through contextual influences by the colour of neighbouring areas outside its classical receptive field, resulting in chromatic induction [5,6]. Computational models based on centre-surround mechanisms, or mimicking the excitatory–inhibitory networks responsible for contextual influences in the primary visual cortex, reproduce induction effects quantitatively [4,11,12] and qualitatively [13].

Two aspects make studying chromatic induction in migraine appealing: the link between migraine and visual phenomena, and the putative excitation/inhibition imbalance in migraine. Migraine is one of the most frequent neurological diseases, affecting approximately 12% of the population worldwide [14,15]. The symptoms of migraine vary from one patient to the other and even from a migraine attack (when the headache occurs) to the other. A large list of factors triggering migraine has been identified, the most frequent of which are hormonal activity, stress, fatigue, lack of sleep, diet, and sensory stimulation [16]. There are several criteria to classify patients with migraine. Migraine patients can be distinguished by the number of days they are in pain every month or taking into account whether they sometimes perceive visual aura before the pain attack or not. Auras consist of visual disturbances such as spots or lines of light that appear in the visual field, usually thirty minutes before pain onset [17]. Typically, an aura starts at the centre of the visual field and spreads to the periphery [18].

Visual perception has been extensively used as a method to explore indirectly brain function in migraine. Differences in visual processing between patients with migraine and control have been found in low level visual processing including contrast sensitivity, orientation discrimination, visual search, masking, motion perception, after-effects and surround suppression [19,20,21,22,23,24,25,26,27,28,29]. Some authors suggest that these differences are caused by cortical hyper-excitability in migraine [30], probably related to an insufficient GABA-ergic inhibition [31,32]. Other authors suggest that these differences are the consequence of impaired inhibitory mechanisms [33,34,35,36,37,38]. Nevertheless, growing evidence points towards an excitatory/inhibitory imbalance in migraine. Regarding colour, several psychophysical studies have found differences in discrimination between migraine and the general population [33,39,40,41]. These studies suggest that migraine sufferers have a different processing in the short-wavelength sensitive (S) cones but no in the long-wavelength (L) and middle-wavelength (M) sensitive cones.

In this study, we explored whether the effects of colour contrast and colour assimilation established with normal observers in the literature are different in migraine. We reasoned that if excitation/inhibition imbalance is a hallmark of migraine, and excitatory–inhibitory mechanisms are at the heart of the contextual influences that cause chromatic induction [5,6], induction phenomena should be different in the migraine and in the normal populations. We formulated the following hypothesis:

**Hypothesis** **1 (H1).** *Chromatic induction is different in the migraine and in the normal populations.*

Besides, as visual phenomena are exacerbated in migraine with aura with respect to migraine without aura [40,42,43], we expected any difference between the migraine group as a whole and the normal population to be magnified in the group of migraine with aura. We therefore formulated a second hypothesis:

**Hypothesis** **2 (H2).** *Differences in chromatic induction between migraine and control, if any, are enhanced in migraine with aura.*

To test these hypotheses, we defined a psychophysical experiment in which we measured the strength of colour induction (both contrast and assimilation) in migraine without aura, migraine with aura and control groups.

## 2. Materials and Methods

### 2.1. Stimuli

**Figure 2:**
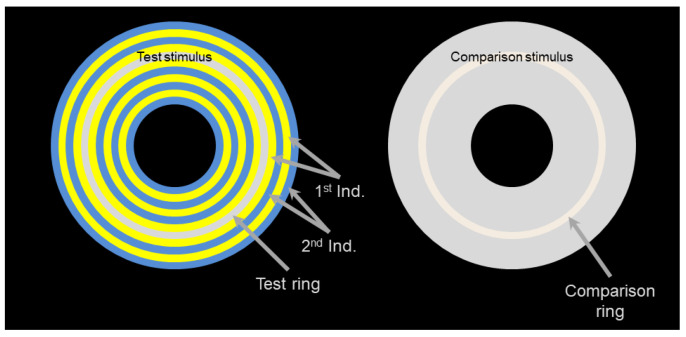
Spatial configuration of the stimuli with 11 stripes. Subjects had to adjust the colour of the comparison ring (**right**) to match the colour of the test ring (**left**). The whole stimulus subtended 17.3×13 degrees of visual angle. The test and the comparison rings had the same size (diameter of 6.91 degrees of visual angle) and their centres were separated by 8.68 degrees of visual angle. The configuration for the stimuli with 17 stripes was in all identical except that the concentric rings were thinner (width of 10.0 arcmin instead of 15.5 arcmin). The colours in the figure are illustrative. Full details on the colours used in the experiment are available in Table 1.

We used the same stimuli as Monnier and Shevell [2] and Otazu et al. [4] because these stimuli have been shown to induce strong chromatic induction effects in control groups, with striped surrounds tending to cause colour assimilation and uniform surrounds tending to cause colour contrast. All the stimuli consisted of a set of concentric rings (see Figure 2, left). The test ring, whose colour had to be matched by the participants when adjusting the comparison ring (Figure 2, right; see ’Procedure’ below), was in the middle of the set. For both striped and uniform stimuli, the area surrounding the test ring was made of two different inducers (the 1st and the 2nd ones, in order of their distance to the test ring). In uniform surrounds, however, the 1st and the 2nd inducers had the same colour. All the stimuli occupied the same area of visual field.

Of the three spatial frequencies considered in Otazu et al. [4], we used the two highest, namely, 11 and 17 concentric rings, corresponding to a width of 15.5 arcmin and 10.0 arcmin, respectively, because high frequencies cause more assimilation than low frequencies [4,44,45]. We defined the same eight colour conditions (four striped and four uniform, see Table 1). Overall, this led to a set of 16 possible stimuli.

### 2.2. Experimental Setup

To generate the stimuli, we used the MATLAB’s Libraries [47] from the Cambridge Research Systems Toolbox (Cambridge Research Systems, Ltd., Rochester, UK). The stimuli were displayed on a 21” SONY GDM-F500R CRT monitor (1024×768 px) calibrated using a ColorCAL colorimeter (Minolta sensor) and the Cambridge Research Systems software. All stimuli were presented through the ViSaGe MKII Stimulus Generator. Participants viewed the stimuli binocularly from a distance of 140 cm (subtending 17.3×13 degrees of visual angle) in a dark room. The responses of the observers were collected using a Logitech© gamepad.

### 2.3. Participants

A total of 21 participants took part in the experiment: 7 (5 females and 2 males, 26.5±7.56 y.o.) had migraine with aura (MA), 7 (3 females and 4 males, 23.57±6.53 y.o.) migraine without aura (MO) and 7 (1 female and 6 males, 25.57±9.40 y.o.) were control subjects with no migraine (C). Migraine subjects were diagnosed according to the criteria of the International Headache Society (IHS). Controls did not suffer neurological symptoms or primary headache satisfying the IHS classification criteria [17]. As inclusion criteria, observers had to have a normal colour vision, as evaluated using the Ishihara’s test [48] and the D-15 Farnsworth Dichotomous test [49], and a normal or corrected-to-normal vision.

All subjects gave their informed consent to participate in the study, which was conducted in accordance with the Declaration of Helsinki. The protocol was approved by the Ethics Committee of our University (Comissió d’Ètica en l’Experimentació Animal i Humana de la Universitat Autònoma de Barcelona, CEEAH-4056).

### 2.4. Procedure

The subjects first adapted to the dark environment of the experimental room during three minutes before the experiment started. For each stimulus, the subjects were asked to match the colour of the comparison ring to the colour of the test ring (see Figure 2). To do so, they could navigate freely through the MacLeod–Boynton colour space using the buttons of the gamepad. Once subjects thought they had found the best possible match, they pressed a button and a blank frame appeared for 5 s to reduce the stimulus after-effect and, next, a new stimulus was presented.

There was no time restriction to do the task, but participants were advised not to take more than one minute per stimulus. The experiment consisted of 6 blocks, 2 per day during 3 days. In each block the series of 16 possible stimuli was presented twice, in random order. In total, the participants therefore did 12 repetitions for each stimulus. Before starting the experiment, the participants did a one-day training session to familiarise themselves with the task and the environment of the laboratory. All the data collected during this training session were discarded from the analyses.

### 2.5. Reading Chromatic Induction in the MacLeod–Boynton Colour Space

**Figure 3:**
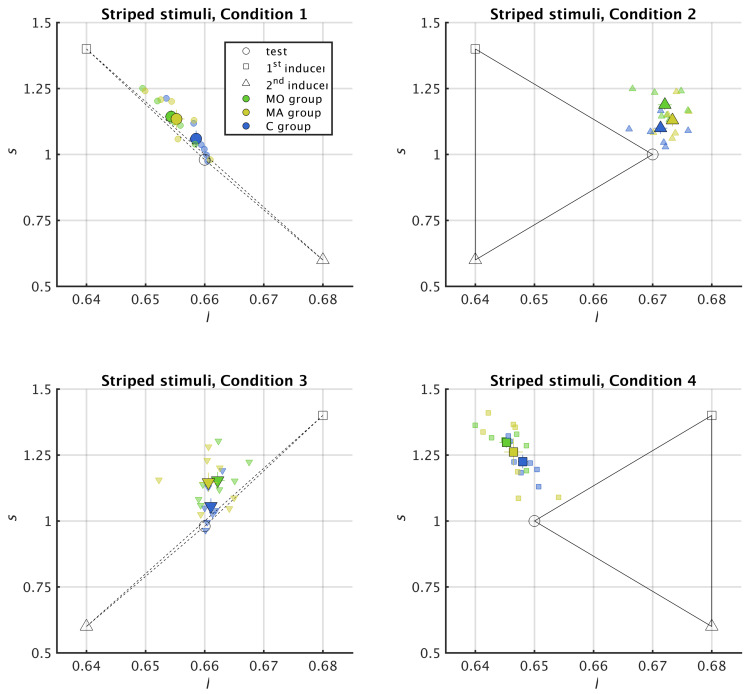
Experimental results for striped surrounds (Conditions 1–4 of Table 1) and 17 stripes represented in the MacLeod–Boynton chromatic space (l,s). The filled symbols of different colours indicate the perceived colours (the chosen values for the comparison ring) for the different groups (migraine without aura, MO, migraine with aura, MA and control, C). The small symbols represent the individual data and the big ones represent the group means. The error bars indicate the standard error of the mean. The open symbols show the colour of the first (squares) and second (triangles) inducers, and of the test ring (circles).

To identify chromatic induction effect, in Figure 3 and Figure 5 we represented the colours involved in each of the 8 colour conditions as well as the colours chosen by the participants in the MacLeod–Boynton (l,s) space. The test colour is represented by an open circle, the colour of the first inducer by an open square and the colour of the second inducer by an open triangle; the colours perceived by the participants are represented by filled symbols with bars showing the standard errors of the mean along each axis. For each trial, if the chosen comparison colour is between the test and the 1st inducer, there is colour assimilation. If instead the test colour is between the comparison and the 1st inducer, there is colour contrast.

### 2.6. Metric for Chromatic Induction

As the *l* and *s* axes of the MacLeod–Boynton colour space are not perceptually comparable, to compare the strength of colour induction among the different stimulus configurations we used a metric for colour induction defined in [12,50] that treats the two axes separately. For each axis *i*, where i=[l,s], we computed the ratio of the difference between the output of the experiment, i.e., the chosen, or ’perceived’, colour, $C_{i}^{c}$, and the test colour $C_{i}^{t}$, and the difference between the colour of the (first) inducer $C_{i}^{s}$ and the test colour $C_{i}^{t}$, as

(1) $$\Delta C_{i}=\frac{C_{i}^{c}-C_{i}^{t}}{C_{i}^{s}-C_{i}^{t}}.$$

The term $\Delta C_{i}$, a scalar value, therefore represents the strength of induction along the axis direction *i*. This metric not only makes comparable the strength of colour induction along the *l* and *s* axes of the MacLeod–Boynton space, but is also sensitive to both colour contrast ($\Delta C_{i}<0$) and colour assimilation ($\Delta C_{i}>0$). More specifically, according to Equation (1), $\Delta C_{i}$ is negative when the chromaticity of the comparison ring $C_{i}^{c}$ shifts away from that of the first inducer $C_{i}^{s}$ with respect to the test colour (the signs of $C_{i}^{c}-C_{i}^{t}$ and $C_{i}^{s}-C_{i}^{t}$ are opposed). By contrast, when $\Delta C_{i}$ is positive the chromaticity of the comparison ring shifts towards that of the first inducer ($C_{i}^{c}-C_{i}^{t}$ and $C_{i}^{s}-C_{i}^{t}$ have the same sign). The metric does not include information about the second inducer because the first inducer only determines the polarity of induction. It is important to note that there is a region below the just noticeable difference (JND) in which no colour change can be perceived and, therefore, no colour induction effect can be measured. We estimated this region (ΔE=1) from the CIELab colour space as this space is approximately perceptually uniform. We then transformed this estimate to the MacLeod–Boynton colour space and to the metric units afterwards (see [12]).

### 2.7. Statistical Analysis

Data were separated according to chromatic condition and spatial frequency. We first removed outlier observations for each observer separately using the interquartile range measure (σ=1.5, [51]) for each arrangement (a chromatic condition and a spatial frequency). To analyse the main effect of group, i.e., migraine status, while taking into account that each observer was presented with each stimulus several times, we used mixed models. Group (alternatively, MO + MA versus C, and MO, MA and C taken separately) was treated as a fixed factor while observer identity was treated as a random factor. The statistical models were fitted using the function *lmer* from the package lme4 [52] in R [53].

To test our hypotheses, we used likelihood ratio tests against a $\chi^{2}$ distribution with degrees of freedom equal to the difference in degrees of freedom between the null model and the model with group (i.e., 1 in MO + MA versus C, and 2 in MO versus MA versus C).

## 3. Results

In agreement with former studies (Monnier and Shevell [2], Otazu et al. [4]), arrangements with striped surrounds and arrangements with uniform surrounds caused distinct induction effects. Below, we first describe the results for striped surrounds and next for uniform surrounds.

### 3.1. Striped Surround

**Figure 4:**
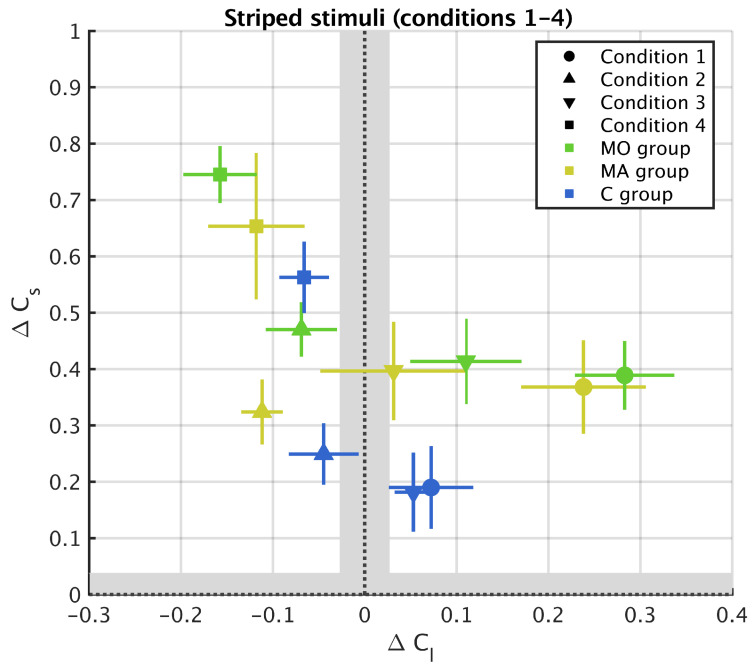
Experimental results for striped surround (Conditions 1–4 of Table 1) represented in the metric units (see Equation (1)). The term $\Delta C_{l}$ measures chromatic induction along the *l* axis of the MacLeod–Boynton colour space and $\Delta C_{s}$ measures chromatic induction along the *s* axis. The bigger |ΔC| the stronger chromatic induction. Positive values of the metric (ΔC>0) correspond to assimilation and negative values (ΔC<0) to contrast. The grey region corresponds to one Just Noticeable Difference. The symbols and the bars respectively represent the mean and standard error of the mean in each group and for each condition.

**Figure 5:**
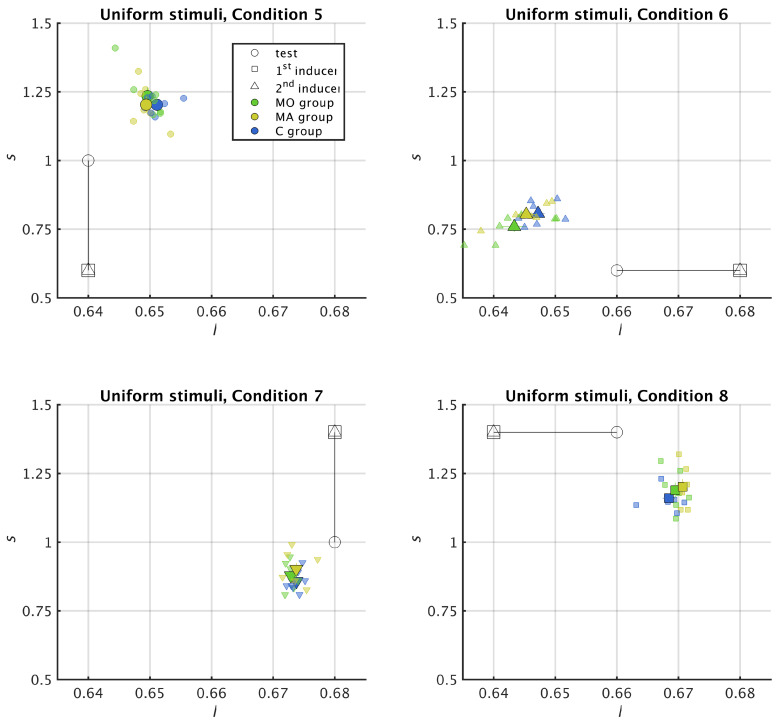
Experimental results for uniform surround (Conditions 5–8 of Table 1) represented in the MacLeod–Boynton chromatic space (l,s). As in Figure 3, open circles represent the chromatic conditions and the filled symbols represent the observed results, with the small ones the individual data and the big ones the group means.

**Figure 6:**
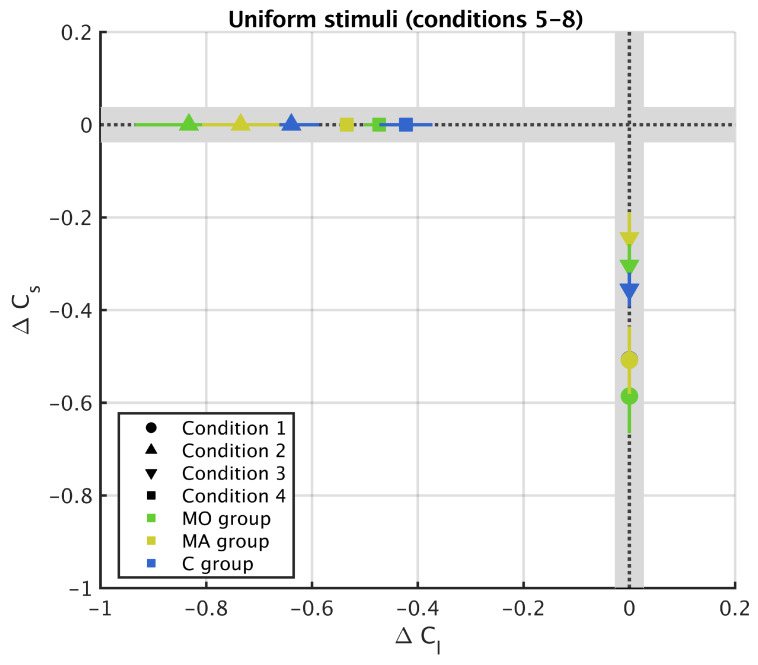
Experimental results for uniform surrounds (Conditions 5–8 of Table 1) in metric units (see Equation (1)) along the two chromatic axes *l* and *s*. The larger the |ΔC|, the stronger the chromatic induction. Positive values show that colour assimilation is occurring and negative ones show that colour contrast is occurring. Grey region indicates the JND region. Symbols indicate the mean value for the different groups and error bars indicate the standard error of the mean.

**Figure A1:**
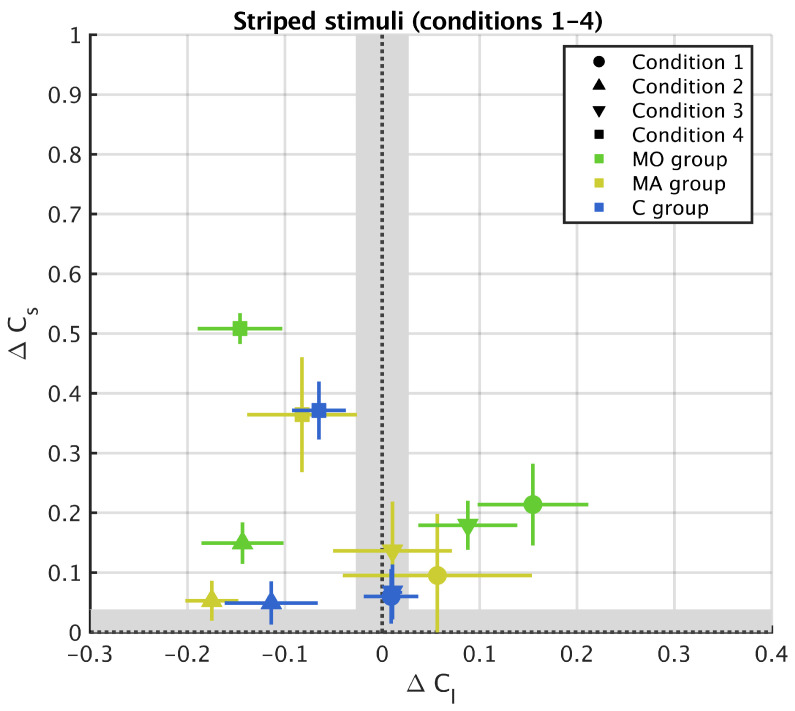
Similar to Figure 4, the colour induction is shown for the three different groups. Here, we present the results for the 11 stripes arrangement.

**Figure A2:**
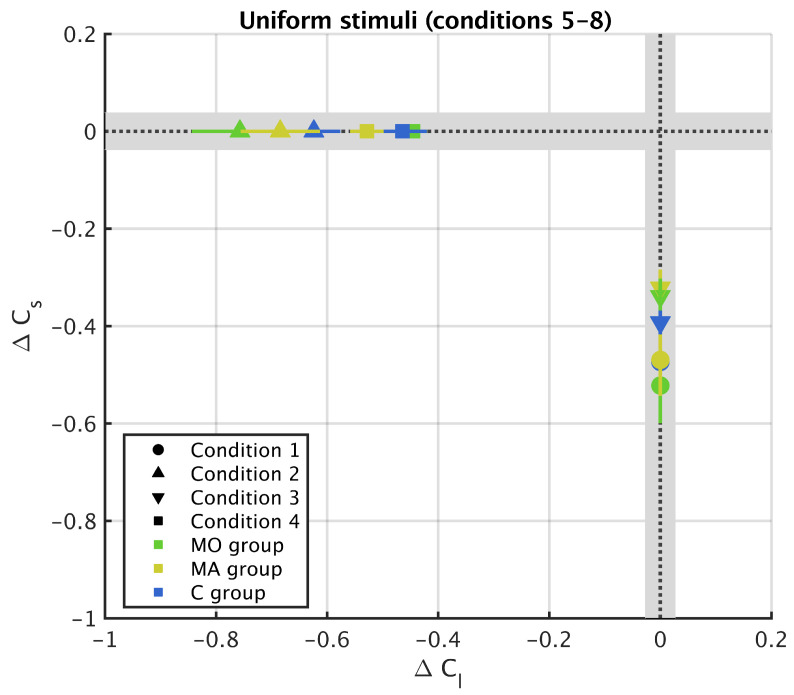
In this figure, we present the psychophysical results for uniform surrounds for stimuli composed by 11 stripes. Results are similar to the ones shown in Figure 6.

We found similar induction effects with 17 and 11 stripes. For brevity, we present here the results for 17 stripes (Figure 3, Figure 4, Figure 5 and Figure 6) and refer to the Appendix A for the results with 11 stripes (Figure A1 and Figure A2).

In Conditions 1 and 3, the stripes surrounding the test ring had different values of *l*, with the *l* value for the 1st inducer greater—and that of the 2nd inducer lower—than the *l* value of the test ring, or vice versa, see Table 1. In Conditions 2 and 4, by contrast, there was no difference in *l* between the 1st and the 2nd inducers. From previous studies (Monnier and Shevell [2], Otazu et al. [4]), we therefore expected to observe colour assimilation along the *l* axis in Conditions 1 and 3 and colour contrast along the *l* axis in Conditions 2 and 4 .

The results supported these predictions. Conditions 1 and 3 caused colour assimilation along this axis (filled circles and filled downward oriented triangles are in the right hand side, i.e., positive $\Delta C_{l}$, quadrant in Figure 4). Conditions 2 and 4 caused colour contrast (filled upward oriented triangles and filled squares are in the left, i.e., negative $\Delta C_{l}$, quadrant in Figure 4). Regarding the *s* axis, in the light of previous studies (Monnier and Shevell [2], Otazu et al. [4]), we expected to observe colour assimilation in all conditions because all stimuli compose a surround with alternating *s* values. In agreement with this prediction, we found that all the perceived colours are in the upper (positive $\Delta C_{s}$) quadrants, showing that colour assimilation is always occurring along the s axis (Figure 4). In line with Hypothesis 1, colour induction was different in the migraine and control groups: both $\Delta C_{l}$, $\chi^{2}=9.19$, p=0.0024, and $\Delta C_{s}$, $\chi^{2}=5.51$, p=0.0189 were higher in migraine than in control. In contrast with Hypothesis 2, however, colour induction was stronger in migraine without aura than in migraine with aura ($\Delta C_{l}$, $\chi^{2}=9.35$, p=0.0093; $\Delta C_{s}$, $\chi^{2}=6.10$, p=0.0475).

### 3.2. Uniform Surround

For all the uniform surrounds (Conditions 5 to 8) the test ring and the inducer are on the same axis of the MacLeod–Boynton colour space (*l* axis for Conditions 5 and 7 and *s* axis for Conditions 6 and 8). In each Condition, the metric for chromatic induction is therefore only defined along one axis, as shown in Figure 6.

We observed colour contrast for all the groups in the four conditions (the values of $\Delta C_{l}$, Conditions 5 and 7, and $\Delta C_{s}$, Conditions 6 and 8, are negative for all groups, Figure 6). In contrast with the stimuli made of striped surround, we did not find any evidence of difference between groups for the uniform surrounds (Figure 5, *l* axis, MO + MA versus C, $\chi^{2}=2.75$, p=0.10; MA versus MO versus C: $\chi^{2}=2.76$, p=0.25; *s* axis, M versus C, $\chi^{2}=0.09$, p=0.76; MA versus MO versus C, $\chi^{2}=0.65$, p=0.72), indicating that the strength of the colour contrast was the same in all groups.

## 4. Discussion

We measured chromatic induction using colour stimuli already defined in the literature. The effects of induction we found were consistent with previous studies: striped surrounds caused chromatic assimilation and uniform surrounds caused chromatic contrast. The novel contribution was to compare chromatic induction in migraine and normal populations, assessing potential differences between migraine with and without aura.

Lateral interactions in the visual cortex are thought to play a central role in chromatic induction [5,6]. As these interactions are underpinned by excitatory and inhibitory mechanisms, and experimental evidence shows a link between excitation/inhibition imbalance and migraine, we expected chromatic induction to be different in migraine and control (Hypothesis 1). Our results suggest that this is indeed the case, at least with one of the two classes of stimuli used in the experiment. When presented with striped stimuli, both chromatic contrast and assimilation were stronger in migraine than in control. Our data did not support any difference between the two groups for uniform surrounds. While our study did not use a continuum of spatial frequencies in the surround but only two types of surrounds (uniform, and high spatial frequencies) to capture a transition in induction effects between uniform and striped surrounds with increasing frequencies [5,6], a possible reason for the difference between the two types of surrounds is that the excitatory–inhibitory mechanisms that define lateral connections were not or weakly activated in uniform surrounds. On the other hand, we observed that the difference between migraine and control groups were consistent along both *s* and *l* axes of the MacLeod–Boynton colour space. As the *s* axis of the MacLeod–Boyton space describes changes in colour involving *S* cones, differences along the *s* axis are in line with studies pointing out that colour perception in migraine people differs whenever *S*-cone processing is involved [19,33,39,54,55]. Our results, however, also supports differences between migraine and control groups in the mechanisms involving *L* and *M* cones.

The prevalence of visual phenomena is higher in migraine with aura than in migraine without aura. We therefore expected a difference in chromatic induction between the control and the migraine groups to be exacerbated in migraine with aura (Hypothesis 2). Our data do not support such a claim. Instead, we found mild evidence of the opposite, namely, that chromatic induction is stronger in migraine without aura than in migraine with aura. A limit of our study is that the experimental sessions took place on different days. The observers with migraine are therefore likely to have done the experiments at different phases of the migraine cycle, which, given our limited sample size, may have driven the difference found between the two migraine groups. According to the literature [56], it is indeed possible that colour induction, as other visual phenomena, changes along the migraine cycle. Future studies of chromatic induction in migraine may monitor the observers’ migraine cycle by, for example, asking observers to complete a migraine diary including migraine attacks, their duration, intensity, and associated symptoms, and running repetitive lab sessions along the migraine cycle. Another limitation of this study is that observers in the migraine group were classified according to whether they had auras or not before migraine attacks. Other criteria, such that the number of days with a migraine attack per month, may correlate better with different strength of chromatic induction.

Although the striped stimuli we defined had symmetric chromaticities with respect to the l=0.66 value, they did not cause symmetric induction effects. For instance, in Figure 3 the colours perceived in Condition 1 lay approximately along the diagonal that joins the inducers. In particular, MO and MA subjects are close to l=0.655. This diagonal displacement means that colour assimilation is occurring in both the *l* and the *s* axes. Instead, Condition 3, which is symmetric with respect to Condition 1, causes assimilation along the *s* axis only; MO and MA values for *l* are close to 0.66. This vertical displacement means that there is colour assimilation only along the *s* axis. Moreover, Condition 4 causes a stronger assimilation in the *s* axis than Condition 2. The dependency between chromatic induction and chromaticity of the inducers we observed is in line with previous studies in which we concluded that colour assimilation does not only depend on the differences in luminance between inducers but also on the chromaticity of the inducers [12,50].

## 5. Conclusions

We measured chromatic induction in migraine and control populations and found enhanced effects of induction in migraine. As induction results from the modulation of the colour of a region by the colour of surrounding regions through lateral interactions, and lateral interactions are underpinned by interactions between excitatory and inhibitory connections, this result is consistent with the excitation/inhibition imbalance found in migraine. Our results, on the other hand, did not support the hypothesis that the difference measured between migraine and control is enhanced in migraine with aura, but gave mild evidence of the opposite.

This study could be useful to assess visual differences between people with and without migraine, and to assess visual differences between the different phases involved in the migraine cycle for people with migraine.

## Figures and Tables

**Table 1:** Colours used in the experiments. Chromaticities are defined in the MacLeod–Boynton colour space, with *s* normalised to unity equal-energy white [46].

**Experiment S (Striped Surround)**									
**Condition**	**Test Ring**			**1st Inducer**			**2nd Inducer**		
	***l***	***s***	***Y***	***l***	***s***	***Y***	***l***	***s***	***Y***
1	0.66	0.98	27.5	0.64	1.40	20.0	0.68	0.60	37.0
2	0.67	1.00	26.0	0.64	1.40	20.0	0.64	0.60	32.0
3	0.66	0.98	27.5	0.68	1.40	22.0	0.64	0.60	32.0
4	0.65	1.00	30.0	0.68	1.40	22.0	0.68	0.60	37.0
**Experiment U (Uniform Surround)**									
**Condition**	**Test Ring**			**Inducer**					
	***l***	***s***	***Y***	***l***	***s***	***Y***			
5	0.64	1.00	26.0	0.64	0.60	32.0			
6	0.66	0.60	34.5	0.68	0.60	37.0			
7	0.68	1.00	29.5	0.68	1.40	22.0			
8	0.66	1.40	21.0	0.64	1.40	20.0			

## Data Availability

Experimental data points can be downloaded from Neurobit’s webpage, accessed on 22 June 2021.

## Sample Availability

Code for generating stimuli, display stimuli and perform data analysis are available from the authors at Neurobit’s webpage, accessed on 22 June 2021.

## Abbreviations

The following abbreviations are used in this manuscript:

C	Healthy migraine-free control
JND	Just Noticeable Difference
M	Migraine
MA	Migraine with aura
MO	Migraine without aura

## Appendix A. Results from 11 Stripes Conditions

The following Figure A1 and Figure A2 show the psychophysical results for 11 stripes configuration. The colour induction effect here is not as big as the one induced in the 17 stripes conditions.
